# Toxicological effects of sublethal microcystin-LR exposure in *Labeo rohita*: histopathological, ultrastructural, immunological, and biochemical impairments

**DOI:** 10.3389/ftox.2025.1658995

**Published:** 2025-10-20

**Authors:** Snatashree Mohanty, Anirban Paul, K. V. Rajendran, Gayatri Tripathi, Pratap Chandra Das, Pramoda Kumar Sahoo

**Affiliations:** ^1^ ICAR-Central Institute of Freshwater Aquaculture, Bhubaneswar, India; ^2^ ICAR- Central Institute of Fisheries Education, Mumbai, India

**Keywords:** microcystin-LR, multi-organ toxicity, detoxification, immunosuppression, Labeo rohita, antioxidant system

## Abstract

**Introduction:**

Microcystins (MCs) are alarming aquatic contaminants having extensive health implications in fish. Despite growing concern, comprehensive studies on long term effects of MCs remain unexplored in *Labeo rohita (rohu)*. This study aims to bridge the gap by investigating the pathophysiological effects of long-term sublethal exposure to microcystin-LR (MC-LR)*,* the most toxic congener.

**Methods:**

Healthy rohu juveniles (mean weight 25 ± 2.1 g), sourced from institute farm were acclimatized for 2 weeks. The sublethal experimental study consisted of two treatments (control group: T0 and one-tenth 96 h-LD_50_ dose group: T1) in three replications (10 fish per tank). The toxic effects were examined after 90 days by analysing histomorphology, ultrastructure, oxidative stress level, serum biochemistry, and the gene expression levels of antioxidant enzyme [superoxide dismutase (SOD) and catalase], immune-related (lysozyme, and immunoglobulin M), pro-inflammatory cytokine (interleukin-1β), apoptosis (caspase 9) and detoxification enzyme [phase I: CYP1A and CYP3A; phase II: glutathione-S-transferase (GST)] genes following standard analytical methods. Statistical analysis was performed using SPSS v. 22.0, IBM software. Parameters were analysed using an unpaired t-test. The results were expressed as mean ± standard error (SE).

**Results:**

MC-LR induced significant histological and ultrastructural alterations including vacuolation, hepatocyte degeneration, disintegration of heterochromatin, loss of nucleolus and mitochondrial swelling. It significantly (*p-value* <0.05) altered the immune and serum biochemical indices. Interestingly, the modulation in the expression of SOD, catalase, GST, CYP1A and CYP3A genes in different organs indicated their involvement in the antioxidant and detoxification process. A significant upregulation of GST expression in all organs signifies its potential as a prominent biomarker other than phase I enzymes.

**Discussion:**

Based on these findings, it is deduced that even sublethal levels of MC-LR can disrupt intrinsic antioxidant defences, immune responses, and detoxification mechanisms in rohu, potentially compromising fish health in natural ecosystems. This is the first report to detail long-term impacts in rohu, elucidating the mechanism of damage induced by MC-LR and also providing valuable insights for environmental monitoring and toxin management.

## Highlights

• Sub-lethal exposure of MC-LR strongly incites cellular and subcellular markers.
• Oxidative stress was identified as a major driver of toxicity.
• Activation of phase I and II molecules rendered partial protection to the organs.
• Pathological consequences overshadow the detoxification process of MC-LR in rohu.

## 1 Introduction

The burgeoning influx of nutrients into the freshwater ecosystem leading to eutrophication has been a worldwide problem. This scenario has accelerated the proliferation of cyanobacterial blooms driven by combined effects of anthropogenic pressure, intensification and global warming. This not only weakens the integrity of the ecosystem but also threatens aquatic lives by secreting secondary metabolic compounds, i.e., cyanotoxins. Microcystins (MCs) are high-risk cyanotoxins, which are potential health hazards causing multi-organ toxicity in fish, amphibians, birds, and humans (Mohanty et al., 2024). MCs inhibit PP1 and PP2A, and protein phosphatases activities leading to hyperphosphorylation of regulated proteins, thus significantly affecting cytoskeleton organization, signaling pathways, metabolism, and cell cycle (Trinchet et al., 2011). The broad toxic effects of MC-LR include hepatotoxicity, immunotoxicity, cardiotoxicity, neurotoxicity, and reproductive toxicity, as documented in various fish species (Falfushynska et al., 2023; Mohanty et al., 2024). However, in-depth literature is limited to the Indian major carp, rohu (*Labeo rohita*), an important freshwater food fish in the Asian continent. Fishes often consume toxic cyanobacteria and may get intoxicated with released microcystin during the algal senescence phase. Hence, fish are accepted as suitable biological models for evaluating aquatic ecotoxicity. Free microcystin concentrations at a very high level of 337.3 mg/kg in fish have been recorded (Magalhaees et al., 2001), which is alarming and needs consistent research attention. Due to its potential toxicity, a limit of 1.0 μg/L and 0.04 mg/kg of body weight/day of MC-LR in drinking water and in fish muscles, respectively has been established by WHO (2011).

The acute toxic effect with a single intraperitoneal injection of MC-LR in rohu revealed an LD_50_ dose of 713 μg/kg with immunosuppression, mitochondrial-mediated apoptosis, oxidative stress, and pathological effects in important organs (Mohanty et al., 2024). The sublethal toxicological studies hold pertinent reasons since water bodies are more frequently exposed to low concentrations of microcystins than lethal doses. Sublethal doses of MCs can cause substantial damage in the intestine, spleen, kidney, muscles, and gills in fish, and exhibit organotropism towards the liver. MCs also induce pathological lesions in the liver, including cytoskeletal disruption, glycogen depletion, hepatic haemorrhage, and necrosis or apoptosis (Malbrouck and Kestemont, 2006; Li et al., 2007; Rodrigues et al., 2022). Aquatic ecosystem is often challenged with relatively low concentrations of MC-LR over extended periods. This type of prolonged exposure has received limited attention primarily because of low mortality rates and a lack of visible changes in fish. However, in field conditions, the detrimental effects of subchronic stress are often more persistent, which are mostly ignored and underestimated (Ernst et al., 2001; Boaru et al., 2006; Mohamed et al., 2015; Zhou et al., 2025). In India, reports on increased eutrophication followed by bloom in freshwater bodies point towards the safety concern of aquatic life and its transfer to the human food chain. For instance, Singh and Asthana (2014) recorded bioaccumulation of MCs more than the safe limit of WHO guidelines in muscles of catfish, which indicates a likely threat to humans on consumption of catfish and strongly recommended routine analyses of MCs in pond water as well as fishes to avoid animal and human intoxication.

Detoxifications of xenobiotics are biological processes that undergo phase I (hydrolysis, oxidation, and reduction) and phase II (conjugation) reactions, which converts high hydrophobic molecules to hydrophilic molecules and their subsequent elimination (Sheweita, 2000). Phase I enzymes belong to the cytochrome P450 (CYP) family and are widely found across various mammals, fish, plants, and microorganisms (Bathe and Tissier, 2019), whereas CYP1A and CYP3A subfamilies serve as biomarkers for aquatic environment contamination (Uno et al., 2012). Similarly, in phase II, GST protects tissues from oxidative stress and is involved in detoxifying xenobiotics. CYP450 and GST enzymes can metabolize and detoxify more than 90% of drugs and xenobiotic contaminants (Landi, 2000; Nelson, 2003). This research work aims to study the long-term (sub-lethal single exposure) effect of MC-LR by determining changes in histomorphology, oxidative stress, non-specific immunity, serum biochemistry, and expression changes at the gene (antioxidants, immune-related, apoptosis and detoxification enzymes) level in rohu, the predominantly preferred, cultured and accepted food fish of Indian sub-continent.

## 2 Materials and methods

Microcystin-LR with a purity of more than 95% was procured from Enzo Life Sciences, USA. A stock solution was prepared following Hipsher et al. (2021) using 2% Dimethyl Sulfoxide (DMSO) as a diluent of MC-LR and stored at −20 °C till further use. All other reagents were of analytical grade and purchased from Sigma (United States).

### 2.1. Experimental animal

Healthy *L. rohita* juveniles (mean weight 25 ± 2.1 g) sourced from ICAR-Central Institute of Freshwater Aquaculture (ICAR-CIFA), Bhubaneswar, India, farm were acclimatized for 2 weeks and fed with commercial feed (28% crude protein) at 3% of body weight. Partial water exchange was performed once per day to ensure a clean and healthy environment. Experimental studies in fish were conducted following the institute’s ethical guidelines. Important water quality parameters such as temperature, dissolved oxygen and pH were recorded at monthly intervals to represent the prevailing experimental conditions. The ranges of water quality parameters recorded across tanks were temperature; 24.0–26.5 °C, dissolved oxygen; 6.8–8.0 ppm, pH; 7.48–8.24.

### 2.2 Sublethal chronic toxicity test

The 96-h median lethal dose (LD_50_) of MC-LR, as 713 μg kg^-1^ was calculated from our earlier study in a static bioassay (Mohanty et al., 2024). The sublethal experiment was conducted using one-tenth of the 96 h-LD_50_ dose, i.e. 71.3 μg kg^-1^ toxin. After acclimatization, sixty fish were divided in to two experimental groups: 1) MC-LR group received an intraperitoneal injection of MC-LR at one-tenth of the 96-hour LD_50_ dose (71.3 μg kg^-1^), and 2) Control group, the non-injected naïve group. Each treatment consisted of three replicates and with each replicate corresponding to a separate tank. The control group was handled similarly to that of the treatment group to rule out any handling stress. Both the control and treatment groups were maintained under identical environmental conditions throughout the experiment. At the end of experiment, on 90th day blood was sampled from nine randomly selected fish from each treatment. Before sample collection, fish were anaesthetized with MS-222 (tricaine methanesulphonate) and a part of the blood was heparinized for further study. The rest of the blood was allowed to clot and centrifuged at 4,000 *g* for 15 min to obtain serum for biochemical and immunological assays. The sera were stored at −20 °C before analysis. Gills, liver, and kidney were collected (in 10% neutral buffer formalin or 2.5% chilled glutaraldehyde in 0.1 M PBS and RNAlater) for tissue-level changes and gene expression study from the same fish just after bleeding.

### 2.3 Histological and histochemical examinations

The liver, kidney and gill tissues were carefully excised and fixed in 10% neutral buffer formalin for 24 h for routine histology using hematoxylin and eosin (H and E) staining followed by microscopic (Olympus BX43) examination. Additionally, periodic acid schiff (PAS) stain of liver sections was undertaken to evaluate the glycogen content of the hepatocytes (McManus, 1948). For the histological study, one fish was sacrificed from each replicate, totalling three fish per treatment. Consequently, each histopathological assessment was performed from three individual fish, each representing an independent replicate.

### 2.4 Transmission electron microscopy (TEM)

TEM study was conducted at the Institute of Life Sciences, Bhubaneswar, Odisha, India. Liver and kidney tissues (treated and control) were pre-fixed and post-fixed with 2.5% glutaraldehyde and 1% osmium tetroxide (OsO_4_) solutions, respectively. Ultrathin sections were prepared using Leica Ultracut UCT ultramicrotome from resin-embedded blocks and mounted on copper grids. Staining of sections was done using uranyl acetate and lead citrate. Imaging was performed using a photon microscope (JEOL, JAPAN) with Nikon and Imager Z1 Zeiss with Axovision software.

### 2.5 Effect on non-specific immunity

The nitroblue tetrazolium (NBT) assay was conducted to measure the potential of phagocytes to generate reactive oxygen species (ROS) termed as respiratory burst activity (RBA) of blood, according to Anderson and Siwicki (1994). Similarly, serum MPO activity (a well-known heme-enzyme released mostly by activated neutrophils) was estimated following the protocol of Quade and Roth (1997), while serum lysozyme assay was carried out as per the protocol of Ellis (1990).

### 2.6 Haematological and biochemical analysis

Wright-Giemsa staining was undertaken following a standard procedure (Mohanty et al., 2024). To study different erythrocytic cellular abnormalities (ECA) in fish, five thin blood smears were made from each fish (fish = 3). Morphological alterations were studied using light microscope. The serum biochemical attributes viz., total protein (TP), albumin, alkaline phosphatase (ALP), glucose, aspartate aminotransferase (AST), lactate dehydrogenase (LDH), and alanine aminotransferase (ALT) activities, were estimated using commercial kits (Tulip, India).

### 2.7 Gene expression analysis

Quantitative real-time PCR was performed using published primers (Table 1). The genes were selected based on our findings in earlier acute toxicity studies (Mohanty et al., 2024) and previous studies in other fish species (Wang et al., 2006; Chen et al., 2016). Briefly, RNA extraction was done from ∼50 mg of tissues following the manufacturer’s protocol. Synthesis of cDNA was carried out using Verso cDNA synthesis kit (Thermo Fisher Scientific, United States) according to the manufacturer’s protocol. The expression of antioxidant [catalase, superoxide dismutase (SOD)], inflammation (IL-1β), apoptosis (caspase 9), innate (lysozyme), adaptive immune-related (IgM) and detoxification genes [(glutathione s-transferase (GST), CYP1A and CYP3A] of MC-LR and control groups were analyzed in kidney and liver tissues using real-time PCR (ROCHE, Switzerland). A 10 μL reaction mixture consists of 5 μL of KAPA SYBR green qPCR mix (Sigma, United States), 0.2 μL of forward and reverse primers for each specific gene, and 2 μL of cDNA template with final volume adjusted to 10 μL using nuclease-free water. β-actin gene was used as an internal control in the real-time PCR reaction run. ΔCq value was calculated by subtracting the individual gene Cq value from the Cq value of the reference gene. ΔΔCq was obtained by subtracting the ΔCq of each sample from the ΔCq value of the selected calibrator. The fold change was calculated using 2^- ΔΔCq^, for each sample in triplicate and represented in bar diagrams (Paul et al., 2021).

**TABLE 1:** Details of the primers used for real-time gene expression study.

Target genes	Nucleotide base sequence (5′–3′)	Optimum annealing temperature (°C)	References
β-actin	F- TTGGCAATGAGAGGTTCAGGT R- TTGGCATACAGGTCCTTACGG	55	Kar et al. (2013)
Catalase	F-ACCTCTACAACGCCATCT R- ATTCCACTTCCAGTTCTCAG	56	Sharma et al. (2018)
CuZn-SOD	F-ACGGTGGACCAACTGATA R-CAAGTCATCCTCCTTCTCAT	56	Parida and Sahoo (2023)
IL-1β	F- GTGACACTGACTGGAGGAA R-AGTTTGGGCAAGGAAGA	58	Dash et al. (2014)
Lysozyme G	F- AAGCAAATTCCCTCGTCGTG R- GGTTCTGGCATCGATATT	50	Mohapatra et al. (2019)
IgM	F-ACGCTTCACCATCTCCA R-AGCCACCGTAGCCTCTT	54	Kar et al. (2015)
CYP1A	F-GACATCAACGCTCGCTTCAG R-TCGTCCAGTTTCCGGTCTTCC	55	Dar et al. (2020)
CYP3A	F-CAGCGAGGAACACACAGAAA R-CCGGAGCCTTATTAGGGAAG	53	Sharma et al. (2021)
GST-mu	F-GAAGAAGAGCAGACGAGAG R-TGTCACCAAGGAAGTTAGAG	52	Parida and Sahoo (2023)
Caspase 9	F- GCCCACACCCAGCGACATCCT R-TTGGGAGACGGCATCATTTAC	65	Giri et al. (2018)

F, forward; R, reverse.

### 2.8 Statistical analysis

Statistical analysis was performed using SPSS v. 22.0, IBM software. Parameters were analysed using an unpaired t-test. The significant differences (p < 0.05) were calculated between the control and treatment groups. The results were expressed as mean ± standard error (SE). Graphical presentations were done using Graph pad prism software (version 8.0, United States). Pearson correlation analysis for biochemical attributes was done using Microsoft Excel (ver. 10). Student’s t-test was applied to test the significance of correlation co-efficient (r) at 5% level of significance. Additionally, the coefficient of determination (*r* ^2^) was also computed. All statistical calculations were carried out using Excel.

## 3 Results

### 3.1 Light and electron microscopic studies

**FIGURE 1:**
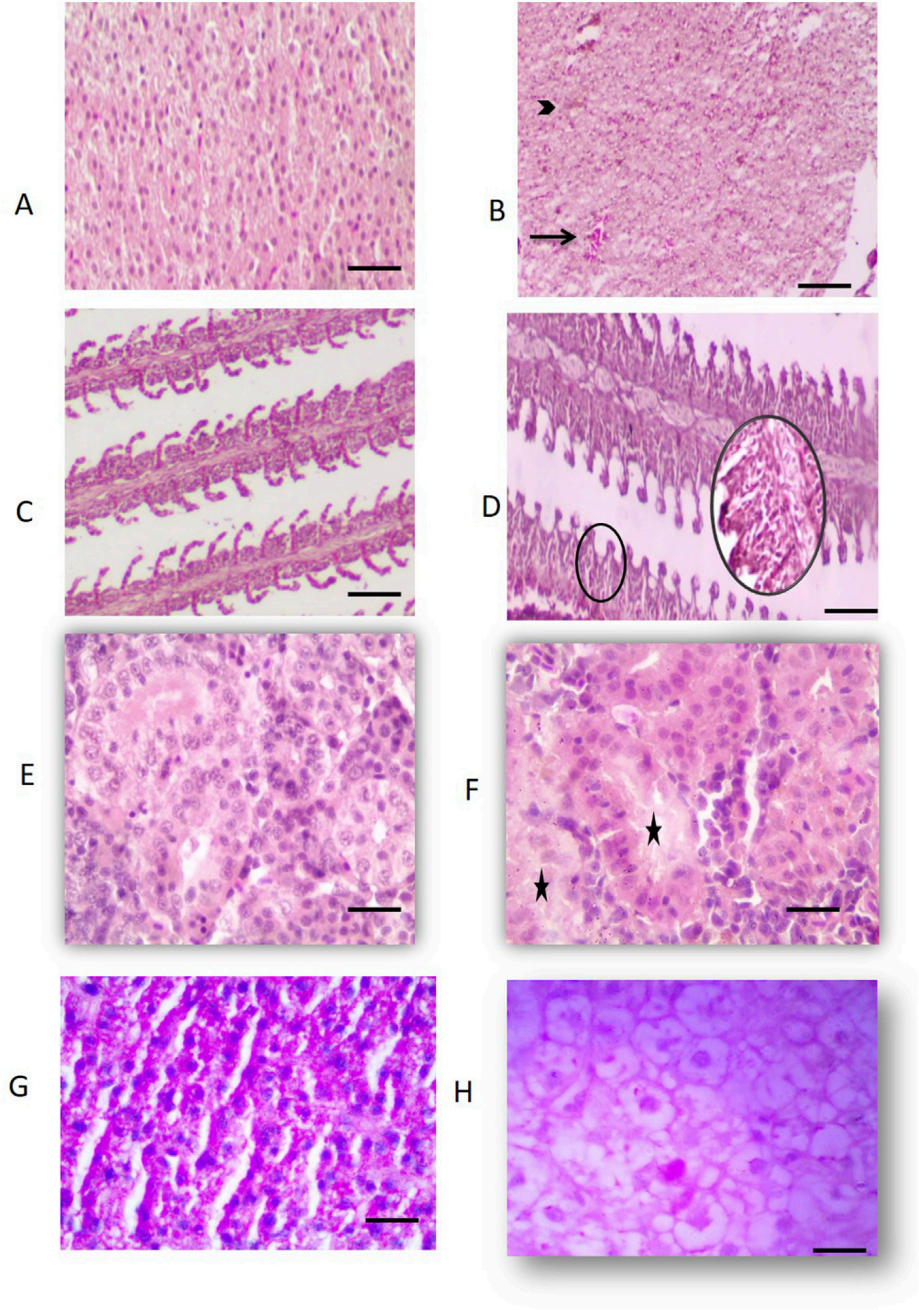
Effects of sub-lethal MC-LR exposure on the histological and histochemical aspects of rohu. **(A)** Representative histological images of liver from the control fish; **(B)** Representative histological images of liver from treated fish; **(C)** Representative histological images of gills from the control fish; **(D)** Representative histological images of gills from treated fish; **(E)** Representative histological images of kidney from the control fish; **(F)** Representative histological images of kidney from treated fish; **(G)** PAS stained control liver showing homogeneously distributed glycogen; **(H)** PAS stained treated liver exhibited depletion of glycogen content prominent vacuolization and necrosis. Black arrow denotes congestion of blood vessels, Black arrowhead indicates hepatocyte cytoplasmic vacuolization. Black circles represent hyperplasia in epithelium and complete fusion of secondary lamellae. Stars indicate massive necrosis of tubules and intertubular haemocytes (Bars represents 25 µm).

Figures 1A–F illustrates the histological changes observed in the liver, gills and kidney tissues of both treated and control groups. The control group of *L. rohita* liver hepatocytes were homogeneously distributed in the parenchyma with the centrally placed spherical nucleus, granular cytoplasm and showed no pathological changes (Figure 1A). However, the treated fish liver sections showed congestion of blood vessels, individualization, and swollen hepatocytes with marked vacuolization (Figure 1B). The gills tissues of control group exhibited an intact epithelium and healthy secondary lamellae, which indicates normal histological architecture (Figure 1C). In contrast, gills from treated fish showed epithelial hyperplasia of epithelial cells at the base of secondary lamellae, leading to complete fusion and curling of secondary lamellae (Figure 1D; indicated by black circles). The microscopic examination of control kidney exhibited normally appearing glomerulus and renal tubules without any lesions (Figure 1E). In contrast, the exposed group of fish kidneys showed massive necrosis of the tubules and lysis of the intertubular haemocyte population, (Figure 1F; indicated by stars). Additionally, PAS-stained control liver tissue showed strong purple-red coloration indicating abundant glycogen (Figure 1G), while chronic exposure led to depletion in glycogen reserves as evident from prominent vacuoles and the reduced intensity of pink colour with hepatocyte swelling as compared to control fish (Figure 1H).

**FIGURE 2:**
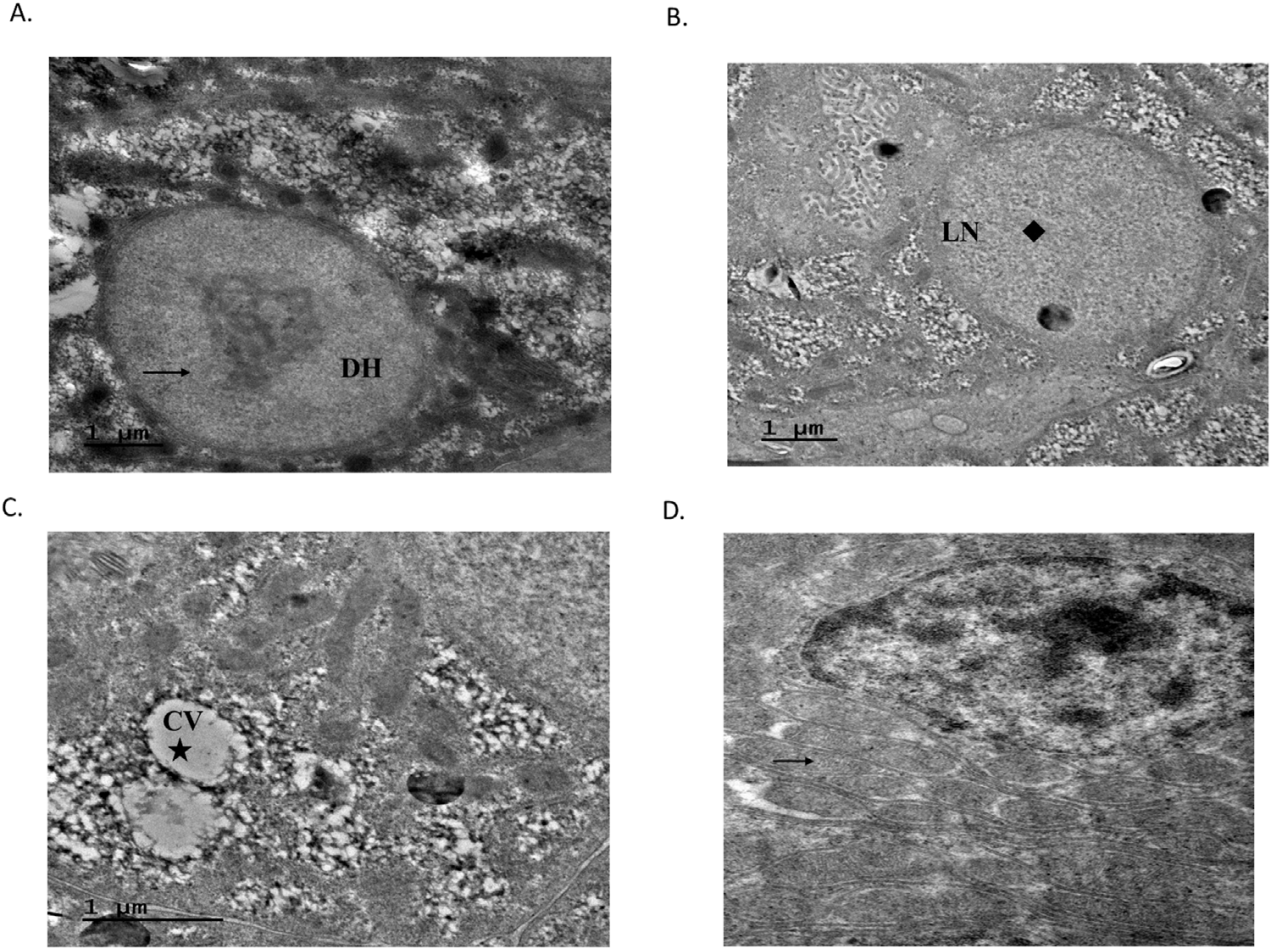
Ultrastructural effects of MC-LR on liver of rohu exposed to 71.3 μg kg^-1^ of MC-LR; **(A)** treated hepatocyte with disintegration of heterochromatin (DH: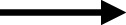); **(B)** loss of nucleolus (LN: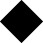) along with stages of degeneration observed; **(C)** cytoplasmic vacuolation (CV: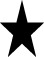); **(D)** increased number of swollen mitochondria (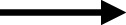).

Transmission electron micrographs of normal liver, kidney, and gill cells showed intact nuclear membranes of different cell types. However, the treated hepatocytes showed disintegration of heterochromatin (DH) and loss of nucleolus (LN) along with stages of degeneration. An increase in number of swollen mitochondriae was recorded under TEM in tubular epithelial cells compared to the control kidney (Figure 2).

### 3.2 Immunological and biochemical assays

**FIGURE 3:**
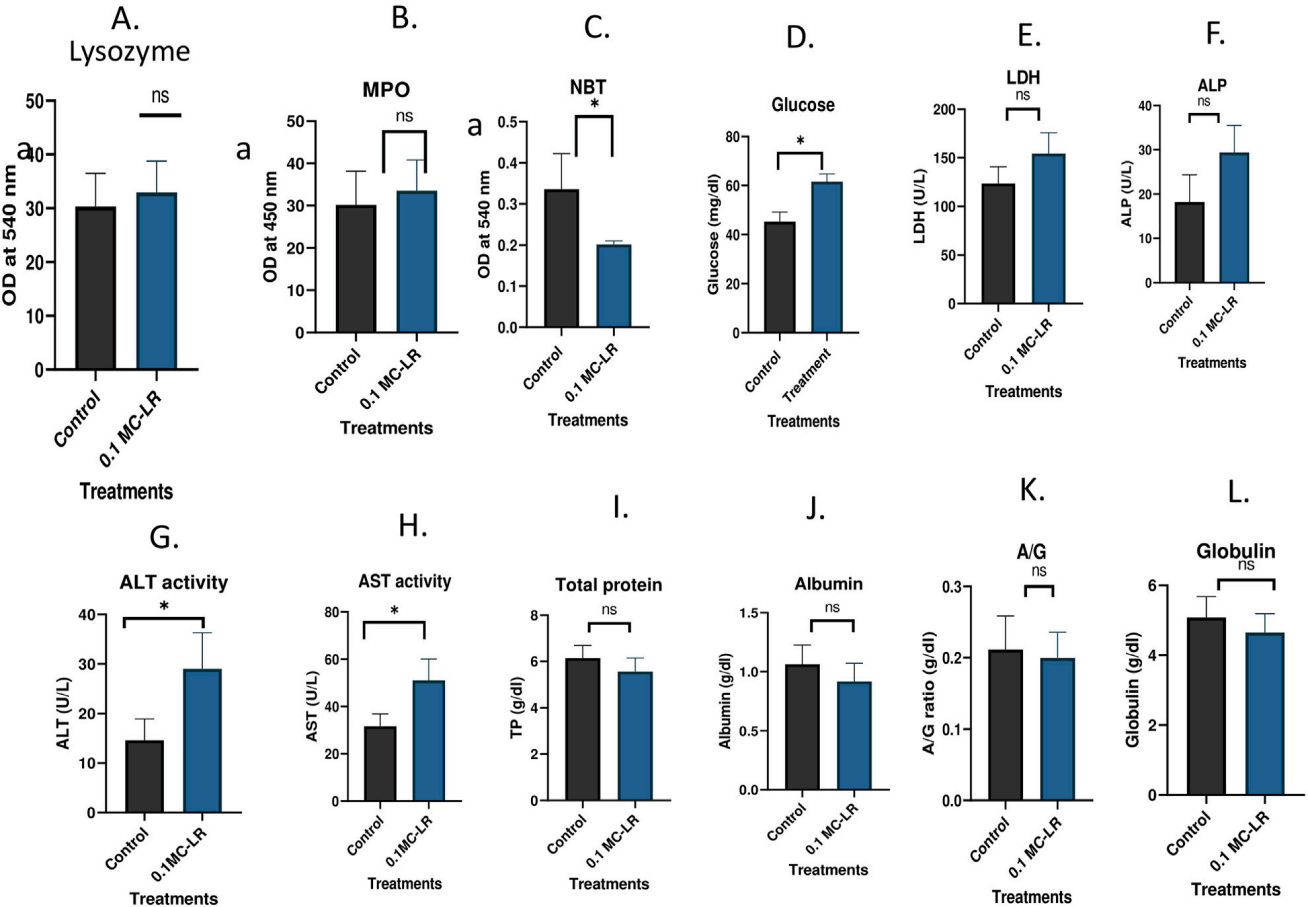
Changes in non-specific immune and biochemical parameters in control and Labeo rohita (rohu) exposed to 71.3 Âμg/kg of MC-LR. The parameters are presented as follows: **(A)** Lysozyme, **(B)** MPO, **(C)** NBT, **(D)** Glucose, **(E)** LDH, **(F)** ALP, **(G)** ALT, **(H)** AST, **(I)** Total protein, **(J)** Albumin, **(K)** A/G, and **(L)** Globulin. Data are expressed as Mean Â± SE. Different letters indicate significant differences (P < 0.05) between the experimental groups. Bars marked with * are significantly different from those of the control group.

**FIGURE 4:**
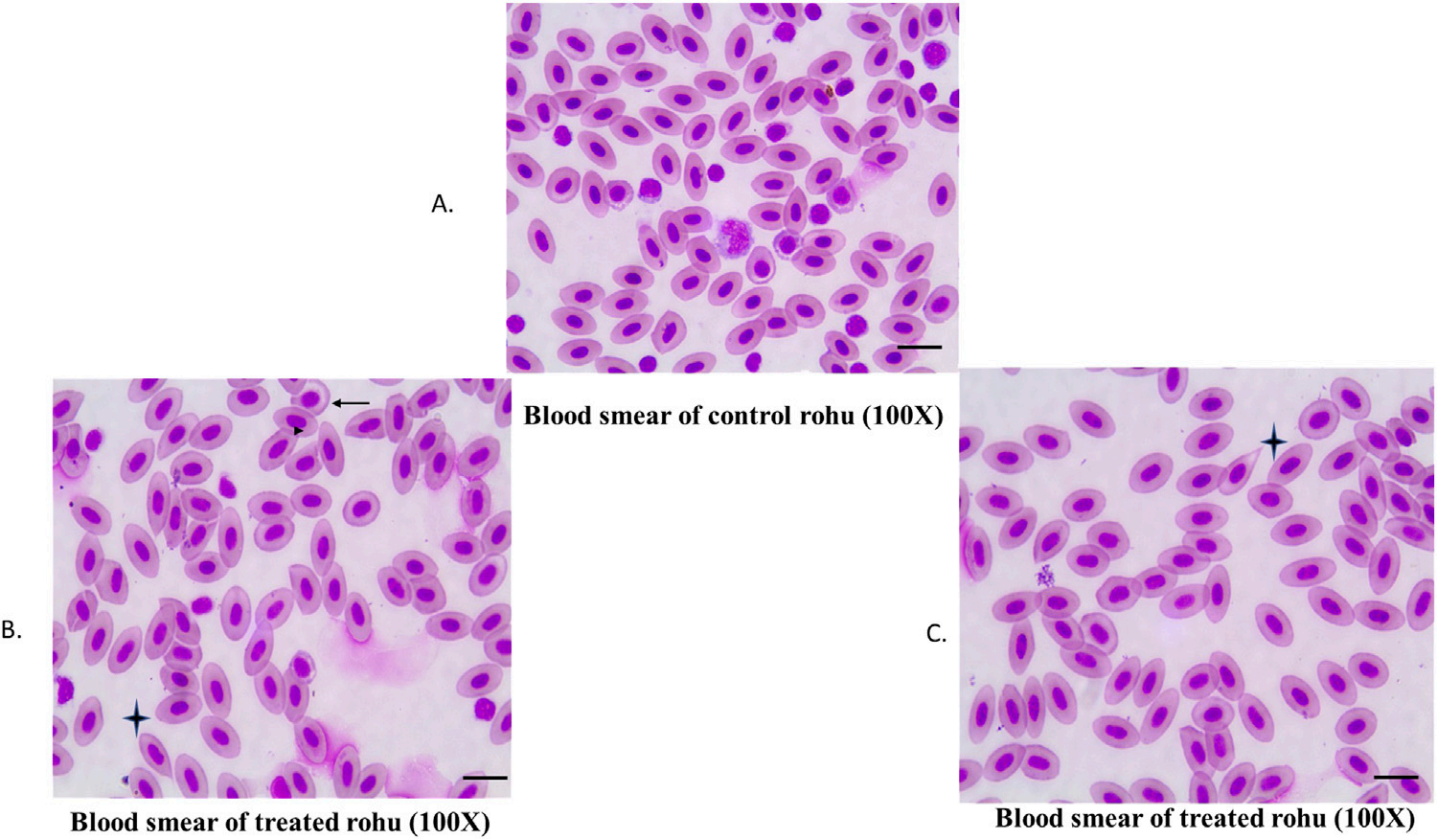
Blood smear showing MC-LR-induced erythrocyte anomalies in rohu with Wright-Geimsa stain. **(A)** Blood smear of control rohu; **(B,C)** represents dacrocyte/tear drop cell (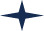); hypochromic cell (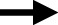) and more numbers of poikilocytes. Bars represents 10 µm.

Non-specific immune and biochemical indices are presented in Figure 3. Unlike that of the control, MC-LR group showed significant (p < 0.05) increase in levels of glucose, ALT, and AST enzyme activities, depicting distinct symptoms of hepatic stress and potential liver damage due to toxin exposure. Other biochemical indices including serum total protein, albumin, globulin, or A/G ratio, LDH and ALP though exhibited an elevated trend but changes were not statistically significant in comparison to control. Regarding non-specific immune parameters, NBT activity was significantly reduced (p < 0.05) in the treated group as compared to the control suggesting a possible suppression of respiratory burst activity in phagocytic immune cells. The blood smears showed MC-LR-induced erythrocyte cellular abnormalities in *L*. *rohita* with poikilocytosis, especially dacrocyte/teardrop cells with prominent blunt ends in erythrocytes and hypochromasia in treated group (Figure 4).

### 3.3 Pearson’s correlation analysis

**FIGURE 5:**
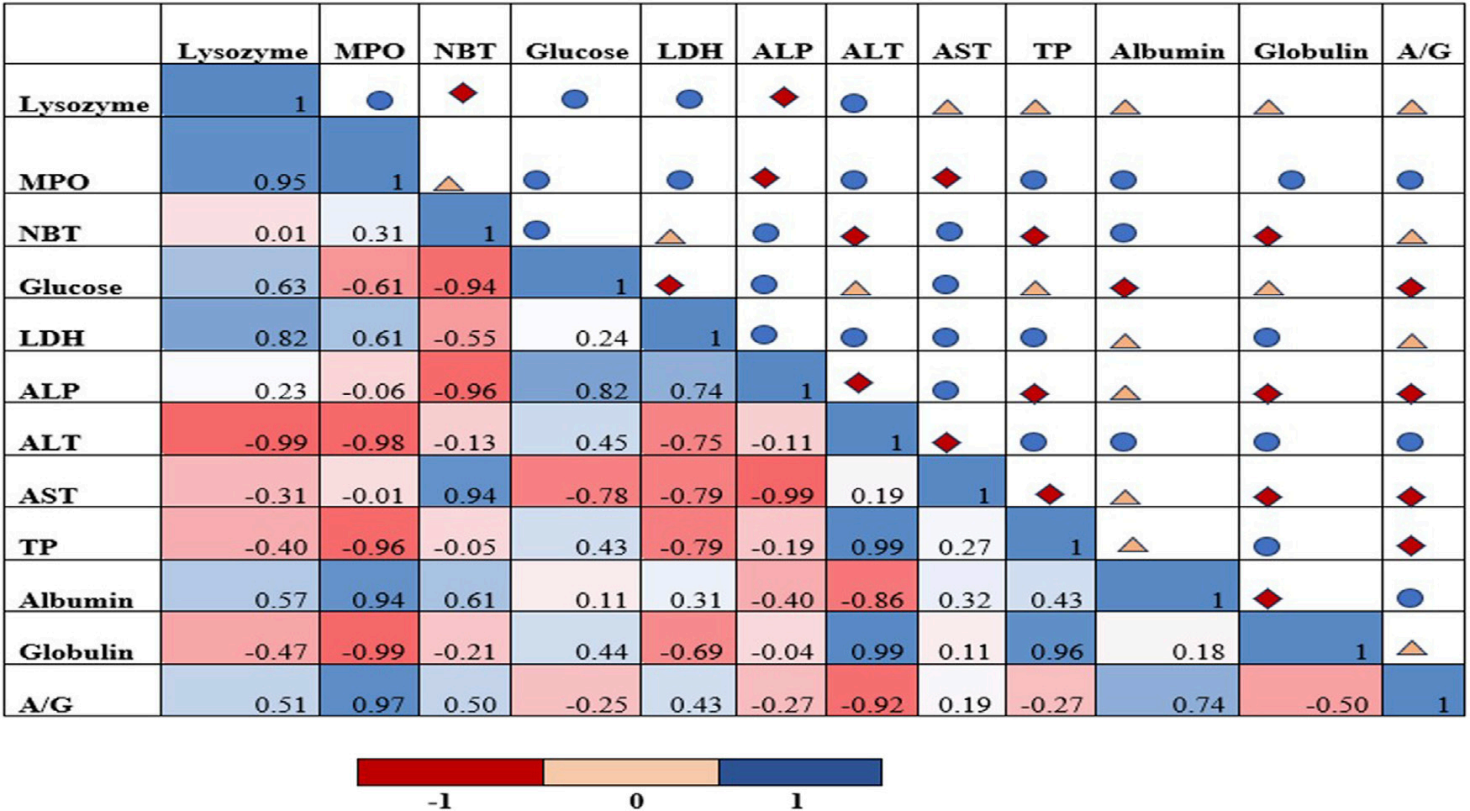
Heat map of Pearson correlation analysis of various biomarkers in rohu injected with sub-lethal dose of MC-LR. Blue coloured circular shapes denote positively correlated variables while red coloured diamond shapes indicate negatively correlated variables. Yellow coloured triangle shapes showed less correlated variables.

Figure 5 depicts a heat map of Pearson’s correlation matrix describing the correlation among analysed immunological and biochemical markers that indicate a close association and their interdependent nature. The results indicated that there is a strong significant positive correlation between lysozyme and MPO (*r* ^2^ = 0.90, p < 0.05) indicating coordinated immune activation. Similarly, TP and globulin were strongly positively correlated (*r* ^2^ = 0.93, p < 0.05), globulin fractions significantly contributes to overall total protein levels in the plasma.

### 3.4 Gene expression analysis

**FIGURE 6:**
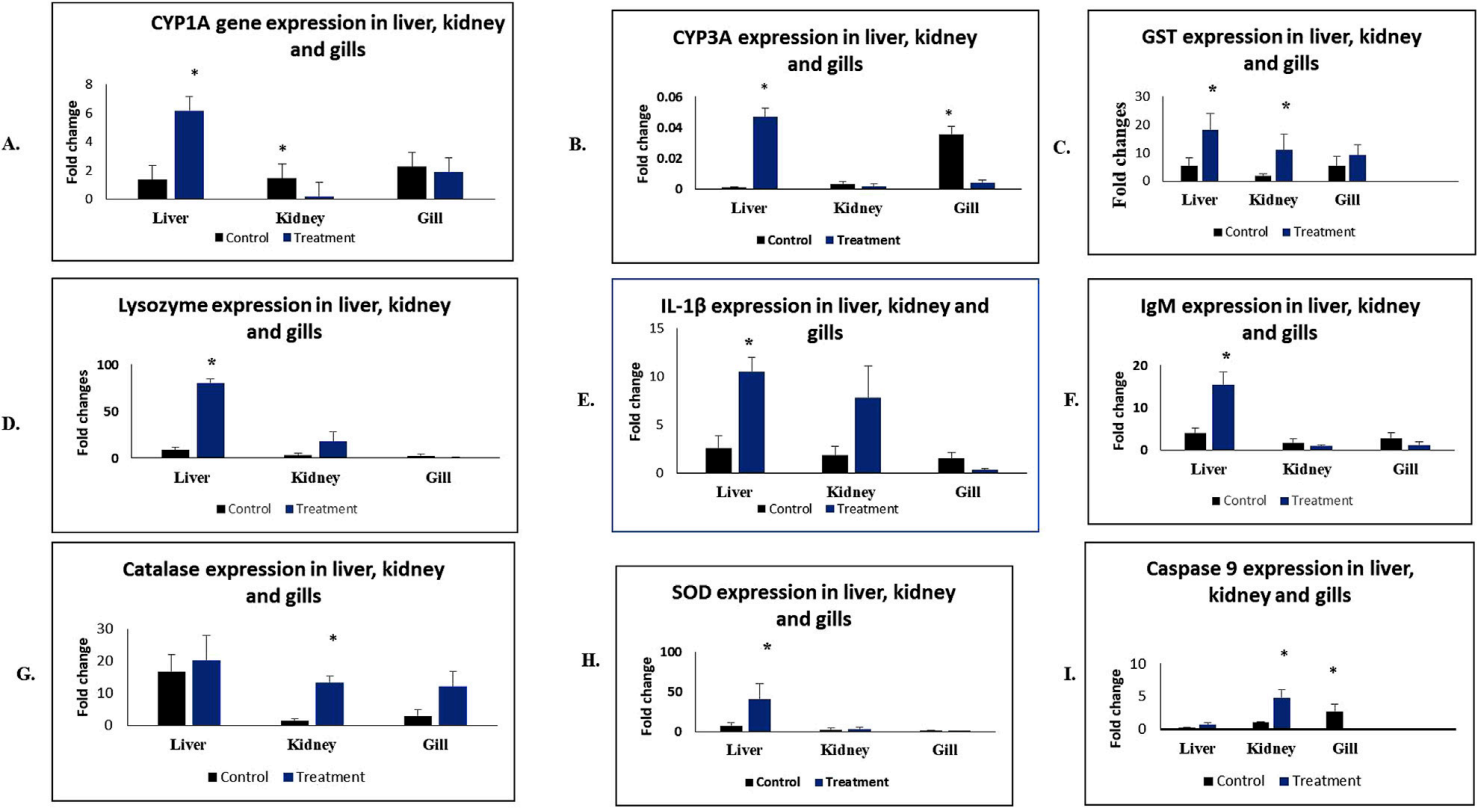
Relative mRNA expression of various genes in the liver and kidney of Labeo rohita (rohu) following exposure to MC-LR. The parameters are presented as follows: **(A)** CYP1A, **(B)** CYP3A, **(C)** GST, **(D)** Lysozyme, Catalase, **(E)** IL-1Î^2^, **(F)** IgM, **(G)** CAT, **(H)** SOD and **(I)** Caspase 9. The data are expressed as mean Â± SE (n = 6).

The expression in mRNA levels of antioxidant, immune-related, inflammatory, apoptosis and detoxification genes of *L. rohita* exposed to MC-LR sub-lethal concentration and control group are presented in Figure 6. Dualistic responses of innate and adaptive systems across the organs were observed. Enhanced mRNA expression of lysozyme (10-fold) was recorded in the liver as compared to the control (p < 0.05). Further, the expression of IL-1β and IgM in the liver was > 4-fold in the treated fish. The expression of caspase 9, an initiator of intrinsic apoptotic pathway, was evaluated in liver, kidney and gill tissues. Kidney and gills exhibited a statistically significant increase in the expression of caspase 9 as compared to the control group (p < 0.05). Transcript levels of phase I (CYP1A and CYP3A) and phase II (GST) enzymes were significantly upregulated (p < 0.05) in liver tissues. CYP1A expression was downregulated in the kidney, while CYP3A expression was reduced in the gills. Interestingly, there was significant upregulation of phase II enzyme, GST expression of 5.8 and 3.3 folds, measured in the kidney and liver respectively (p < 0.05). Additionally, the expression of SOD and CAT were more pronounced in kidney and liver (p < 0.05) respectively.

## 4 Discussion

Fish serves as an exceptional model for ecotoxicological studies, and *L*. *rohita*, being highly preferred in the culture system, can elucidate anti-MC-LR responses happening in nature, especially in tropical conditions. The unique poikilothermic nature of fish exhibits dynamic body physiology with respect to environment. Consequently changes in water quality parameters are expected to influence the body physiology and in turn might affect the response pattern to any toxicant. However, the water quality parameters viz. temperature, dissolved oxygen and pH recorded during the study were well within the ideal range as reported by Jena and Das, (2011), ensuring a stress free environment for the fish. This indicates in our study that, the pathophysiological responses exhibited by rohu, an Indian major carp is mostly associated with the effects of toxicant MC-LR. In the present study, we used a naive control because our previous work (Mohanty et al., 2024) included a DMSO control and did not show any significant changes compared to the naïve group in rohu. Hence, observed outcomes are mostly MC-LR induced and not influenced by the solvent used for its administration. However, the importance of including appropriate solvent controls, such as DMSO or saline, during each individual experiment to ensure reproducibility and to absolutely rule out any potential confounding toxic effects of the solvent should be considered.

Histological and ultrastructural alterations indicate important signatures to elucidate xenobiotic/contaminant-induced changes in an aquatic ecosystem. MC-LR-treated fish exhibited clear vacuolization in hepatocytes and loss of parenchyma followed by necrosis under sublethal exposure. This vacuolation possibly reflects an imbalance between the rate of synthesis of substances in the parenchymal cells and the rate of their release into the systemic circulation (Gingerich, 1982). PAS staining also adds to the toxicity studies in reading cellular changes. Periodic acid oxidizes the glucose residues to produce aldehydes, which further react with the Schiff reagent, leading to a strong purple-magenta colour. A significant reduction in glycogen content, vacuolization, and cellular necrosis was observed in the treated liver with PAS staining. Aligned with the present results, carp gavaged with a single sublethal dose of *M. aeruginosa* cells described similar kinds of tissue-level alterations (Fischer and Dietrich, 2000). Lesions in liver tissues are the results of disturbances in metabolism and oxidative stress, which are most often associated with steatosis (lipid accumulation), protein synthesis inhibition, and energy depletion (Sanchez et al., 2019). The link between xenobiotics and the kidney is inevitable on account of the various fundamental roles viz., excretion and homeostasis played by it. The nephropathy effects in response to MC-LR ranged from dilatation of the Bowman’s capsule in the exposed group and massive necrosis of the tubules and lysis of the intertubular haemocyte population. Fish gills are major respiratory organs for gaseous exchange, excretion of nitrogenous waste products, osmoregulation, and acid-base regulation; hence, these are extremely sensitive to xenobiotics and pollutants (Uchida et al., 2000). Treated fish gills showed curling of secondary lamellae and hyperplasia of epithelial cells at the base of secondary lamellae, leading to complete fusion and separation of epithelium from the vascular layer. Electron microscopic examination revealed nuclear and cytoplasmic changes in hepatocytes and kidney tissues. TEM analysis of the control liver showed a normal appearance of hepatocytes with a centrally placed nucleolus, while treated liver hepatocytes displayed heterochromatin disintegration with loss of nucleolus followed by degeneration of the cells. Cytoplasmic vacuolation of hepatocytes along with necrosis was prominently recorded. Complementary evidence of damages/lesions in the liver, kidney, and gill describes MC-LR as a multi-organ toxin. These cellular and subcellular pathological lesions in the liver, kidney, and gills probably have interfered with normal metabolic and respiratory functions, leading to compromised health. These kinds of dysfunctions in fish exposed to MC-LR are similar to those described elsewhere (Shi et al., 2015).

Environmental contaminants significantly influence the innate immune system and so as fish disease resistance, often assessed by measuring innate immune molecules, i.e., complement, lysozyme and cytokines in response to xenobiotics/pollutants. Humoral molecules, especially lysozyme, can substantially protect gram-positive and negative bacteria in the presence of complement molecules. Nevertheless, monitoring lysozyme activity in fish blood on exposure to environmental contaminants is one of the sensitive biomarkers of environmental contamination (Bols et al., 2001). Phagocytosis in fish is a key non-specific cellular defense mechanism of phagocytes like polymorphonuclear leucocytes, mononuclear cells and macrophages (Low and Sin, 1995). NBT reduction test measures the respiratory burst activity of phagocytes is an integral component of innate immunity. Hence, compromised NBT activity in our study represents a trend of immunosuppression accompanied by lower proinflammatory cytokine production. These cytokines play a major role in the toxicodynamics of MC-LR. Specifically, IL-1β is a key pro-inflammatory cytokine produced predominantly by macrophages in response to tissue damage. Its upregulation indicates the initiation of inflammatory responses during liver injury (Hamsa and Kuttan, 2009; Strowig et al., 2012). Hence, an increased mRNA expression of the IL-1β gene induced by MC-LR in the liver and kidney may indicate altered immune response.

Pathophysiology of fish is assessed by the alterations in biochemical parameters that indicate cell and tissue dysfunction as potential impacts of environmental contaminants. The metabolic function of liver enzymes transaminases, including ALT and AST are important biomarkers to study the healthiness of fish. Significant increase in levels of glucose, ALT and AST compared to the control group depict distinct symptoms of hepatic stress and toxic effects in exposed fish (Alarape et al., 2024). AST plays a crucial role in glutathione biosynthesis and gluconeogenesis in hepatocytes (Ellinger et al., 2011), and its increased activities are linked to mitochondrial disruption associated with intense hepatitis. Cholestasis is a condition of liver dysfunction that impairs bile flow and its retention in the liver. Cholestasis has also been reported to be associated with heavy cyanobacteria bloom events in bighead carp (Qiu et al., 2009). The significant rise in serum biochemical markers viz., ALT, AST, indicated extensive liver damage, as also evident in histological examinations. Similar changes in these parameters have been documented in *Carassius auratus* (Zhang et al., 2008), *Hypophthalmichthys molitrix* (Li et al., 2019) and *Clarias gari*e*pinus* (Alarape et al., 2024).

The strategy to encounter the toxicogenic stress is enhancing the activity of metabolic enzymes and energy investment. This extra demand for energy simultaneously depletes glycogen level in the liver (as observed in the PAS stain) under environmental stress aligned with the significant increase in glucose level in the serum. Notably, these biochemical parameters are interlinked and strongly indicate alterations in carbohydrate metabolism. Blood proteins have vital roles in humoral immune response and in maintaining fish physiological balance. Albumin, globulin and albumin to globulin (A/G ratio) are often used as key diagnostic markers of liver health and immunity. However, their declined activities following MC-LR contamination suggest immunosuppression, and disrupted protein biosynthesis due to liver damage. Similarly, exposure to contaminants like microplastics (MPs) and cadmium has also caused a substantial decrease in blood protein levels in common carp (Banaee et al., 2019) and cage-cultured barramundi (Vazirzadeh et al., 2024).

Interestingly, a sublethal dose of MC-LR in *L*. *rohita* has demonostrated two-way immunotoxicity, i.e., immunosuppression and immunomodulation. This possibly depicts the dynamics of cells to overcome toxicity stress. Significant upregulation of lysozyme and IgM in liver indicates a stimulated innate immune system indicates active participation of liver in MC-LR toxic stress for sustenance. Conversely, reduced NBT activity indicates possible suppression of respiratory burst activity in phagocytic immune cells reflecting a degree of immunosuppression. This phenomenon has been well elaborated by Zhao et al. (2024) in the *Sinocyclocheilus grahami* model to study the immunotoxic effects of *Microcystis aeruginosa* (MaE) and PHS (one of the main components of the MaE). This kind of immunomodulatory potency of cyanotoxins, including both immunostimulatory and immunosuppressive effects, has also been noted by Sierosławska (2010). Nucleated fish erythrocytes participate in immune function through gas exchange, immune gene expression, pathogen recognition, and pathogen clearance (Burgos-Aceves et al., 2021; Ortega-Villaizan et al., 2022). Hence, erythrocyte anomalies noticed in our study indicate poor immune function and oxygen carrying capacity. Moreover, poikilocytosis (abnormal shape of erythrocytes) is a fundamental and emerging biomarker that intricately relates to the underlying physiological status of fish associated with various environmental stressors (Bardhan et al., 2024), including hypoxia, toxins, and temperature changes. Diagnostic values of these poikilocytes during the toxicity study are very important, aiding in health assessment. Our study observed more poikilocytes, especially dacrocyte/teardrop cells (erythrocytes with blunted tips) and hypochromic cells. Herein, we infer that sublethal exposure to MC-LR in *L*. *rohita* resulted in erythro-morphological alterations, possibly a sign of anaemia potentially compromising the fish’s immune system. Similar findings were reported by Islam et al. (2020), who observed erythrocytic alterations under varied concentrations of chromium exposure, supporting our inference.

Biological data sets are often multivariate in nature and difficult to interpret. In this context, drawing correlations among variables through standard statistical methods is essential to quantify the relationship and their interdependent nature. The heat map of Pearson correlation analysis details high/strong, weak, and negative correlation among various parameters represented in varied intensity of blue-coloured circular shapes, red-coloured diamond shapes, and yellow-coloured triangular shapes, respectively. These biomarkers are interlinked and distinguish the intoxicated group as stressed, unlike the control. This tool also helps us to establish association between immunological and biochemical indices. Our analysis revealed a strong positive correlation between lysozyme and MPO as well as between total protein and globulin, indicating coordinated immune and physiological responses under stress conditions. For instance, Similar kinds of interrelated and interdependent behaviours were recorded in *L. rohita* following sub-lethal chronic malathion (Elathion^®^) exposure (Swain et al., 2022; Das et al., 2024). Additionally, Elizalde-Velazquez et al. (2023) documented analogous responses in *Danio rerio* subjected to Bisphenol-A exposure. The interplay and variation of these parameters possibly reflect physiological adaptations of fish at observed time point.

Oxidative stress is an important driver in MC-LR pathogenesis in fish. The intricate balance between the generation and the removal of reactive oxygen species is maintained by a healthy biological system, failing which yields accumulation of the reactive oxygen species (ROS), which is generally detrimental, termed as oxidative stress. ROS can cause damage to the biomolecules like proteins, lipids, and DNA, leading to the onset of various diseases (Wang et al., 2018). Antioxidant enzymes comprise of superoxide dismutase (SOD), catalase (CAT), and glutathione peroxidase (GPx) exhibit protective mechanisms by converting these ROS to harmless molecules (Farombi et al., 2007). SOD catalyzes the dismutation of superoxide (O_2_^−^) to O_2_ and H_2_O_2,_ which is further detoxified by CAT and GPx to H_2_O and O_2_ (Pan et al., 2018). In our study, the MC-LR toxicity has triggered the expression of antioxidant (SOD and CAT) genes as an indicative of oxidative stress response. Increased SOD activity in our study possibly leads to the conversion of O_2_^−^ to H_2_O_2,_ and increased CAT activity subsequently metabolize H_2_O_2_ to O_2_ and H_2_O as part of its intricate antioxidant adaptive response aiming to mitigate oxidative stress and promoting recovery. Similar observations were reported by Ma et al. (2018) with Paraquat stress in common carp. Moreover, the protective and immunomodulatory connection of these antioxidant molecules discovered in *L*. *rohita* by Parida and Sahoo (2023) has added more understanding to our study.

The expression of GST, CYP1A and CYP3A genes were selected for our study to assess their roles in dextoxification post-MC-LR exposure in *L*. *rohita*. Further, we probed more into transcriptional expression of these detoxification enzymes, which showed upregulation of both the phase I (CYP1A, CYP3A) and phase II (GST) enzymes in the liver, possibly indicating their involvement in MC-LR metabolism, and also signified the liver as the most tolerant and active organ for detoxification. The results indicate upregulation of these enzymes or upstream promoters by MC-LR, possibly mediated by oxidative stress. However, to further confirm the actual participation of theses markers in the detoxification process, in-depth functional study is necessary to draw conclusion. With regard to Phase I enzymes, CYP1A expression was downregulated in the kidney, and CYP3A in the gills accompanied by an upregulation of phase II enzyme, GST possibly revealing the role of these two organs towards oxidative stress management rather than metabolic-degradation. Many xenobiotics, including heavy metals, dioxins and PAH derivatives, β-naftoflavonas have also negatively influenced CYP1A activity in fish tissues (Gravato and Santos, 2002). The differential expression may provide evidence of tissue-specific expression of CYP in *L*. *rohita*. Similar transcriptional trends have been observed against triclosan and paraquat stress in rohu and common carp, respectively (Sharma et al., 2021; Ma et al., 2018). Since CYPs’ activation can produce excess ROS leading to oxidative stress (Zheng et al., 2019; Jiaxin et al., 2020), the body boosts up antioxidant enzymes, such as SOD, CAT, and GST for neutralization of free radicals, that supports our findings in MC-LR exposed rohu.

Literature demonstrates that, MC-LR detoxification involves an integral process of MC-LR conjugation with glutathione (GSH) mediated by GST increases solubility of MC-LR for its elimination (Ming et al., 2019; Sabatini et al., 2015). In our study, MC-LR exposure in *L*. *rohita* significantly upregulated GST mRNA expression in the liver and kidney, indicates more biotransformation via GSH conjugation to form less toxic, soluble compounds that prevent cellular lesions. A similar increase in GST activity was reported by Li et al. (2005) and Bieczynski et al. (2013) in *Misgurnus mizolepis* and *Odontesthes hatcheri* fed with *M. aeruginosa*. Our findings suggest that the transcriptional response of phase II enzyme GST might be a potent player in MC-LR detoxification in *L*. *rohita* across all organs. Previous studies have demonstrated organ-dependent toxicity of MC-LR in different fish species. For instance, Chen et al. (2016) revealed varied pathological changes in spleen, gut and gills of zebrafish induced by MC-LR. Hence, in-depth studies are needed to understand the diverse resilience and response of the organs against MC-LR toxicity. This will help to know the tissue-dependent toxicodynamics during MC-LR intoxication, which might be related to the conserved physiological function of each organ.

Caspase 9 plays a critical role in MC-LR-induced apoptosis through the mitochondrial pathway (Zhang et al., 2022; Mohanty et al., 2024). Its expression significantly increased in kidney of the treated group. Interestingly, TEM revealed a noticeable increase in the number of mitochondria in the treated kidney, suggesting more involvement of mitochondria in toxicity process. Moreover, the fold change of caspase 9 was more remarkable in the kidney than in the liver, which means mitochondrial membrane potential in the kidney is more disrupted. Mitochondria invariably take a unique role in the process of apoptosis regulation, and microcystin-induced oxidative stress can potentially affect the mitochondrial membrane potential (Ding et al., 2000). Oxidative stress is a potential inducer of apoptosis. Hence, more caspase 9 (apoptosis marker) expression in the kidney of treated fish in our study may be deliberated and linked towards higher oxidative stress evident from less availability of antioxidant enzymes like SOD and CAT in this organ as noticed in the expression analysis. In contrast, the liver with a significant increase in SOD and CAT activity and robust detoxification system, could potentially protect the liver from oxidative stress, hence, showing less expression of caspase 9. Multiple reasons are associated with the induction of oxidative stress in our present study, *i.e.,* i) the metabolism process of MC-LR; ii) activation of CYP molecules; and iii) the imbalance between generation; and mobilisation of ROS molecules; and iv) affected mitochondrial membrane integrity, which might be the plausible explanation for the initiation of MC-LR toxicity cascade. Our study demonstrated structural and functional impairments of multiple organs along with an underlying toxic mechanism in a freshwater Indian major carp, rohu, in response to MC-LR at a sublethal dose.

## 5 Conclusion

The present study provides preliminary insights in to ecotoxicological effects of sublethal and single long-term exposure of MC-LR in freshwater fish *L*. *rohita* using various biomarkers. Sublethal intoxication with MC-LR has led to various degenerative changes in the liver, kidney, and gills at cellular, subcellular, and molecular levels. Interestingly, varied responses from the liver, kidney, and gills, including immune suppression, compromised antioxidant system, induction of inflammation, and apoptosis, indicate differential resilience patterns of different organs to counteract sublethal exposure to MC-LR toxicity. The observed upregulation of Phase I (CYP1A and CYP3A) and Phase II (GST) gene expressions in various tissues suggests that these genes may be transcriptionally responsive to MC-LR exposure. However, further functional studies are required to confirm whether these enzymes are directly involved in MC-LR metabolism in *L*. *rohita*. The liver displaying maximum expression for all these enzymes indicates its highest detoxification potential. The present study flags enormous ecological threats of an alarming aquatic contaminant MC-LR, in *L*. *rohita* at sublethal level, emphasizing urgent research attention and scientific validation. Future investigations involving fish from algal infested water bodies could provide an applied perspective to the ecotoxicological impacts. Moreover, exploring multiple lines of defence in contaminated fish will deepen our understanding about the stress induced by algal blooms. Given that, these challenges are inevitable in aquaculture practices, adopting a holistic approach is essential for sustainable aquaculture.

## Data Availability

The original contributions presented in the study are included in the article/Supplementary Material, further inquiries can be directed to the corresponding author.
