# Supplementary figures and images for: Toxicological effects of sublethal microcystin-LR exposure in Labeo rohita: histopathological, ultrastructural, immunological, and biochemical impairments
Source: Front Toxicol. 2025 Oct 20;7:1658995. doi: 10.3389/ftox.2025.1658995 (PMC12580125; doi:10.3389/ftox.2025.1658995)

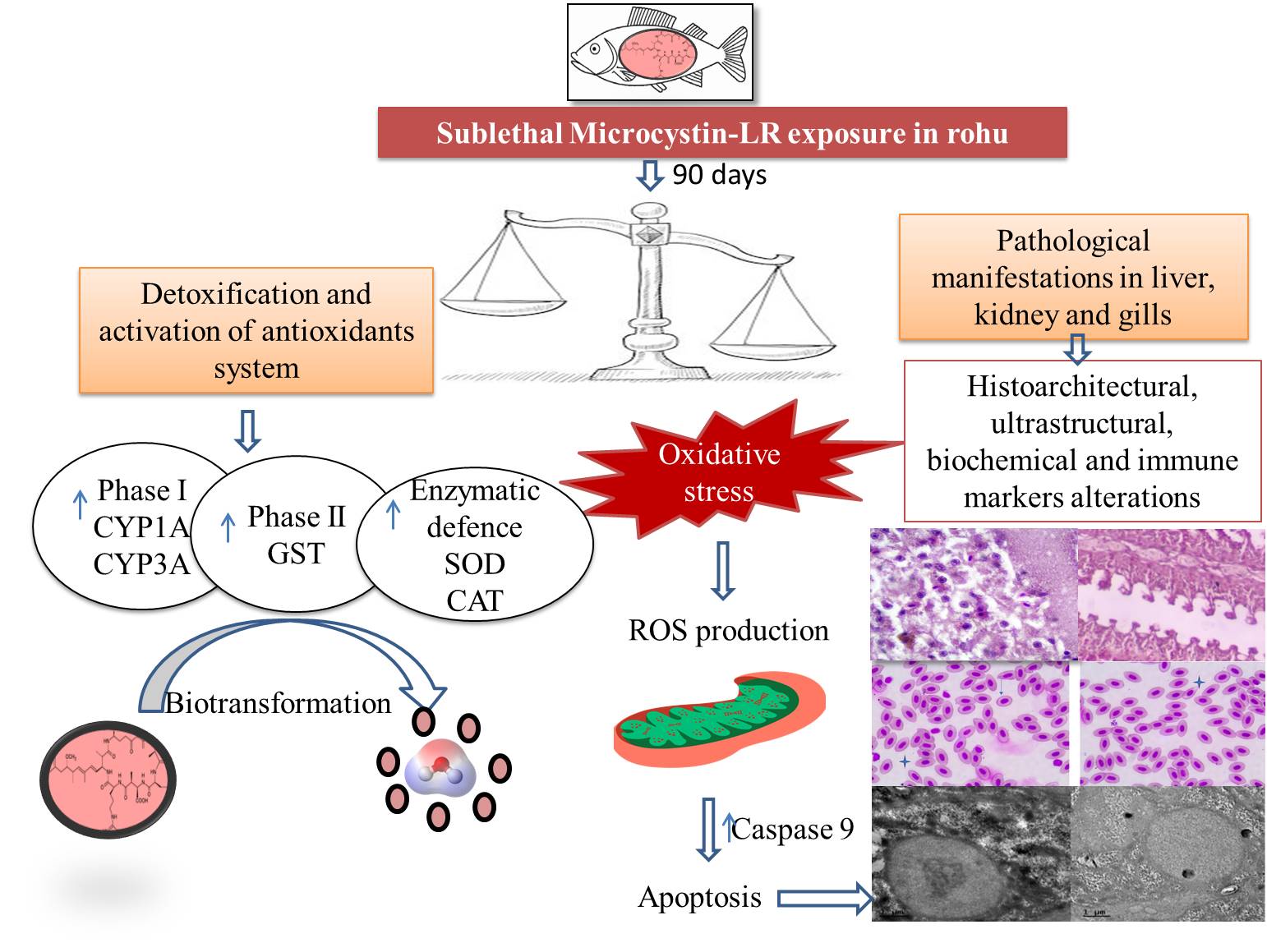

Supplement: Supplementary file 1 [file Image1.jpeg]
